# Growth of Epitaxial ZnSn_x_Ge_1−x_N_2_ Alloys by MBE

**DOI:** 10.1038/s41598-017-12357-9

**Published:** 2017-09-20

**Authors:** Amanda M. Shing, Yulia Tolstova, Nathan S. Lewis, Harry A. Atwater

**Affiliations:** 1 Department of Materials Science, California Institute of Technology, Pasadena, California, 91125 USA; 2 Division of Chemistry and Chemical Engineering, California Institute of Technology, Pasadena, California, 91125 USA; 3 Department of Applied Physics, California Institute of Technology, Pasadena, California, 91125 USA

## Abstract

ZnSn_x_Ge_1−x_N_2_ alloys are chemically miscible semiconductor compounds with potential application as earth-abundant alternatives to In_x_Ga_1−x_N. Preparation of ZnSn_x_Ge_1−x_N_2_ thin-films by reactive RF sputter deposition yield low-mobility, nanocrystalline films. In contrast, the growth of ZnSn_x_Ge_1−x_N_2_ films by molecular-beam epitaxy (MBE) on c-plane sapphire and GaN templates is described herein. Epitaxial films exhibited 3D growth on sapphire and 2D single-crystal quality on GaN, exhibiting substantial improvements in epitaxy and crystallinity relative to nanocrystalline sputtered films. Films on sapphire were n-type with electronic mobilities as high as 18 cm^2^ V^−1^ s^−1^, an order of magnitude greater than the 2 cm^2^ V^−1^ s^−1^ average mobility observed in this work for sputtered films. Mobility differences potentially arise from strain or surface effects originating from growth techniques, or from differences in film thicknesses. In general, MBE growth has provided desired improvements in electronic mobility, epitaxy, and crystal quality that provide encouragement for the continued study of ZnSn_x_Ge_1−x_N_2_ alloys.

## Introduction

In_x_Ga_1−x_N alloys are widely used in light-emitting diodes (LEDs) and sensors, because alloying enables tuning of the band-gap energy, **E**_g_, within the range set by the two binary compounds, InN (**E**_g_ = 0.69 eV) and GaN (**E**_g_ = 3.51 eV)^1^. The large (~10%) lattice mismatch between InN and GaN results in phase separation for In-rich alloys, limiting the ability of In_x_Ga_1−x_N alloys to cover the full visible spectrum^2^, especially the green wavelength range that is important for efficient solar energy conversion.

ZnSn_x_Ge_1−x_N_2_ alloys are emerging nitride compounds with electronic structures similar to those of the well-characterized In_x_Ga_1−x_N alloys. ZnSn_x_Ge_1−x_N_2_ alloys made by reactive RF sputtering have band gaps between 1.8–3.1 eV^3^, which encompasses the visible spectrum, thereby suggesting potential applications as photovoltaic absorber materials, LEDs, or optical sensors. Sputtered ZnSn_x_Ge_1−x_N_2_ alloys have also demonstrated stability against phase segregation throughout the alloy series, which constitutes a significant advantage relative to In_x_Ga_1−x_N alloys^3,4^. Tunability of the structural and optoelectronic properties of the ZnSn_x_Ge_1−x_N_2_ alloy series from 0 ≤ x ≤ 1 is a prerequisite for multi-color LEDs and high-efficiency multijunction solar cells. In addition, ZnSn_x_Ge_1−x_N_2_ alloys are composed of relatively earth-abundant elements; specifically, the least abundant element in ZnSn_x_Ge_1−x_N_2_ alloys is Ge (1.5 mg kg^−1^), which is about six times more abundant in the Earth’s crust than In (0.25 mg kg^−1^)^5^.

Sputtered ZnSn_x_Ge_1−x_N_2_ films have exhibited low electronic mobilities^6^ and trap states from defects^7^, hindering the development of high-performance ZnSn_x_Ge_1−x_N_2_ devices based on this alloy series. Inferior electron transport, and the absence of room-temperature luminescence, may be due to the <200 nm crystallite sizes of the sputtered films, estimated by the Debye-Scherrer framework.^[SI]^ The lack of photoluminescence observed in the films, which were predicted to have direct band gaps by various calculation methods and experimentally generated Tauc plots, is also consistent with the presence of defects that prevent radiative recombination from the conduction band to the valence band in these materials. Annealing and sputtering at higher temperatures (>350 °C) have been explored as routes to larger grain sizes, but crystallites remained <200 nm.^[SI]^ To overcome the limitations of sputtered films, specifically to generate higher mobilities and radiative recombination yields, and to reduce defects and impurities, we have explored film growth by molecular beam epitaxy (MBE).

Plasma-assisted MBE growth of ZnSnN_2_ on cubic yttrium-doped zirconia substrates at 400 °C results in monoclinic ZnSnN_2_^8^. Reflection high-energy electron diffraction (RHEED) and x-ray diffraction (XRD) patterns of such ZnSnN_2_ films indicated crystalline film growth; however microscopic images of the film structure revealed the presence of metal islands on the surface of the films.

We report herein the synthesis of ternary ZnSnN_2_ and quaternary ZnSn_x_Ge_1−x_N_2_ films grown by MBE on c-plane sapphire as well as on wurtzite-GaN templates on sapphire. The films have been characterized structurally and optoelectronically using RHEED, XRD, transmission-electron microscopy (TEM), Raman spectroscopy, ellipsometry, and Hall measurements. Fabrication details for MBE ZnSn_x_Ge_1−x_N_2_ films for 0 < x ≤ 1 are provided in the Supplemental Information.

**Figure 1:**
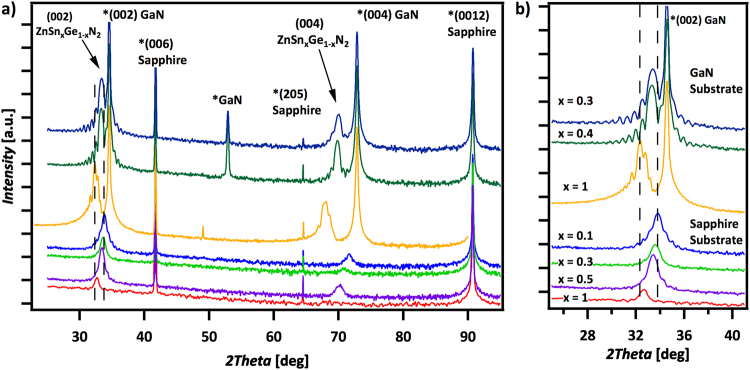
(**a**) XRD patterns of MBE grown ZnSn_x_Ge_1−x_N_2_ on (001) c-sapphire for various compositions. The exclusive presence of the (002) peak indicates that the films are oriented. (**b**) The presence of periodic Pendellösung oscillations on the (002) peak indicates sharp interfaces between the film and substrate as well as pseudomorphic growth that is characteristic of heteroepitaxy.

Figure 1 shows the XRD patterns obtained for ZnSnN_2_ and for several compositions of ZnSn_x_Ge_1−x_N_2_ grown via MBE on c-plane sapphire or GaN substrates. The exclusive observation of (002) ZnSn_x_Ge_1−x_N_2_ peaks and their corresponding higher order reflections for all of the stoichiometries investigated indicated that the films with wurtzite-derived structures were oriented to the substrate. The periodic Pendellösung oscillations on the (002) peak resulted from films with uniform strain and sharp interfaces between the films and substrates^9^, and are consistent with pseudomorphic growth and heteroepitaxy. Growth of ZnSnN_2_ (ZnSn_x_Ge_1−x_N_2_) on sapphire gave rise to weaker Pendellösung oscillations compared to ZnSnN_2_ (ZnSn_x_Ge_1−x_N_2_) films grown on GaN, implying rougher interfaces, arising from the ~20% lattice mismatch between sapphire and ZnSnN_2_ and consistent with the relatively small mismatch, <5%, between the GaN and ZnSnN_2_ lattices^10,11^.

**Figure 2:**
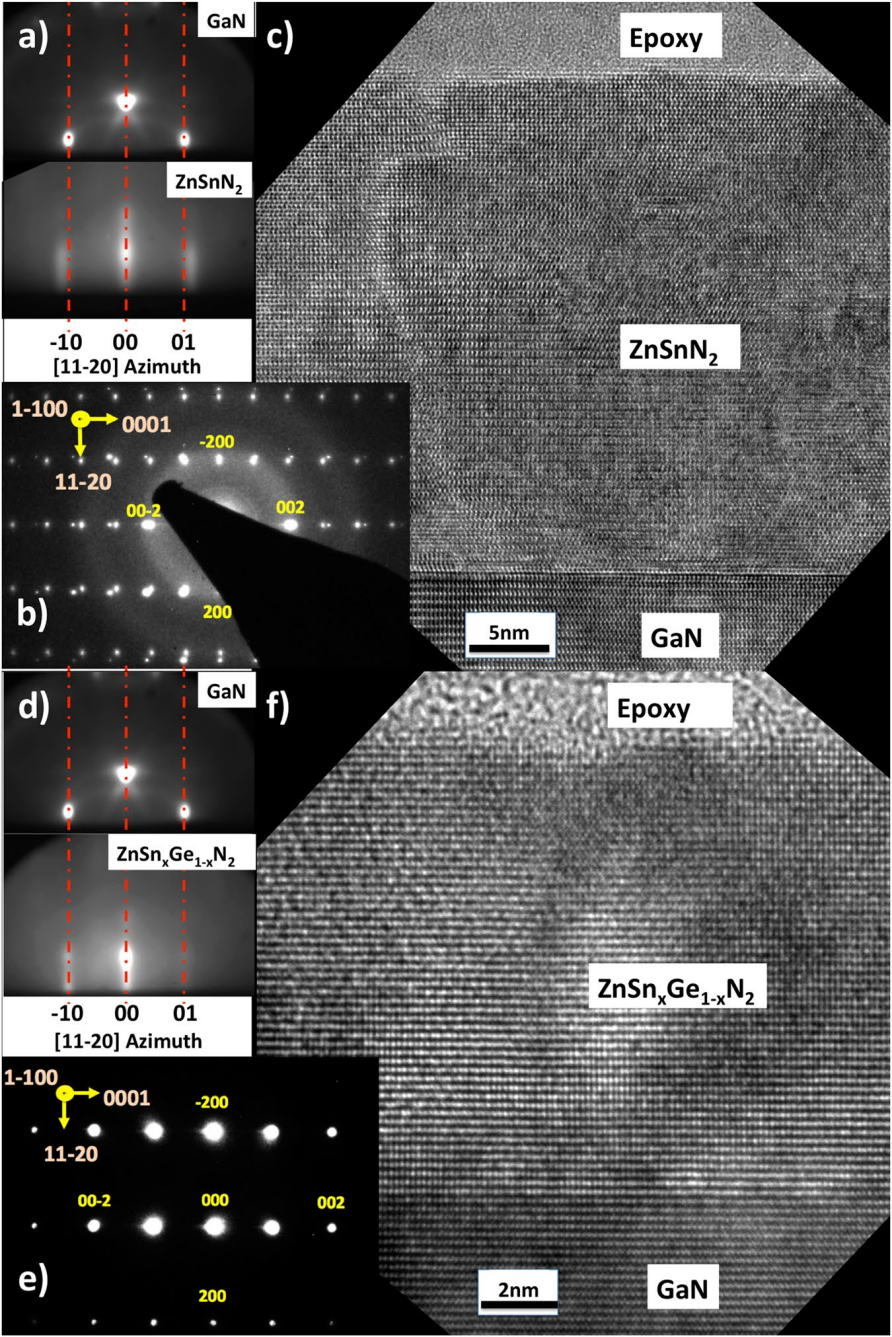
*In-situ* RHEED data for (**a**) ZnSnN_2_ and d) ZnSn_x_Ge_1−x_N_2_ grown on sapphire or GaN substrates for the [11–20] azimuthal directions. Streaked patterns indicate smooth 2D film surfaces, and differences in planar spacing are evident from misalignment of the streaks^27^. Selected-area electron diffraction (SAD) of (**b**) ZnSnN_2_ (x = 1) and e) ZnSn_x_Ge_1−x_N_2_ (with x = 0.3) on GaN. The substrate reflections are clearly separated from the ZnSnN_2_ reflections, showing the lattice mismatch, whereas the ZnSn_x_Ge_1−x_N_2_ reflections sit on the substrate peaks^28^. The rings observed in the ZnSnN_2_ SAD and typical of amorphous structures are from the epoxy applied to the sample. Diffraction spots are labeled for the orthorhombic ZnSn_x_Ge_1−x_N_2_. High-resolution transmission-electron micrographs (HRTEMs) of the GaN substrate and (**c**) ZnSnN_2_ and (**f**) ZnSn_x_Ge_1−x_N_2_ interfaces, exhibiting clean epitaxy. The large-scale variation in contrast present in the HRTEMs indicates strain in the ~20–30 nm films.

RHEED patterns (Fig. 2a,d) for ZnSnN_2_ and ZnSn_x_Ge_1−x_N_2_ films grown on GaN substrates via MBE provided further evidence of epitaxy, smooth interfaces, and lattice mismatch. The streaks apparent in the RHEED images of films on GaN indicated that the films were macroscopically smooth, whereas RHEED patterns for films grown on sapphire substrates (Figure S1) were characterized by intense spots indicative of three-dimensional island growth^12,13^. This difference in growth mechanism is attributable to the corresponding lattice mismatch with the substrate in each case. The relative mismatch between the substrate lattices and nitride alloys of varying Ge content can be seen by a comparison of the RHEED patterns. The interplanar spacing of ZnSnN_2_ at the [11–20] azimuth is less matched to the GaN substrate spacing than to the ZnSn_x_Ge_1−x_N_2_ interplanar spacing (Fig. 2a,d).

Selected area diffraction (SAD) images (Fig. 2b,e) also illustrated the lattice mismatch between the nitride films and GaN substrates. For ZnSnN_2_, the SAD image showed a clear separation between the ZnSnN_2_ reflections and the relatively brighter reflections from the GaN substrate, whereas the ZnSn_x_Ge_1−x_N_2_ reflections were effectively coincident with the substrate peaks, implying lattice-matched films. The positions of the reflections also indicated that the films were hexagon-on-hexagon aligned with the substrate^14^. The amorphous rings in the ZnSnN_2_ SAD background can be attributed to the epoxy applied to the sample. Diffraction spots are labeled for the orthorhombic ZnSn_x_Ge_1−x_N_2_.

Figure 2c and f show high-resolution TEM (HRTEM) images of interfaces of ZnSnN_2_ and ZnSn_x_Ge_1−x_N_2_ with GaN. Cross-sections exhibited few notable defects at the interface or throughout the films, and film surfaces were specular, indicating continuous, smooth films, heteroepitaxy, and single-crystalline morphology. The variations in contrast apparent in the ZnSnN_2_ and ZnSn_x_Ge_1−x_N_2_ layers indicated that strain was present in films of 20–30 nm thickness grown via MBE at substrate temperature settings of 250 °C.

**Figure 3:**
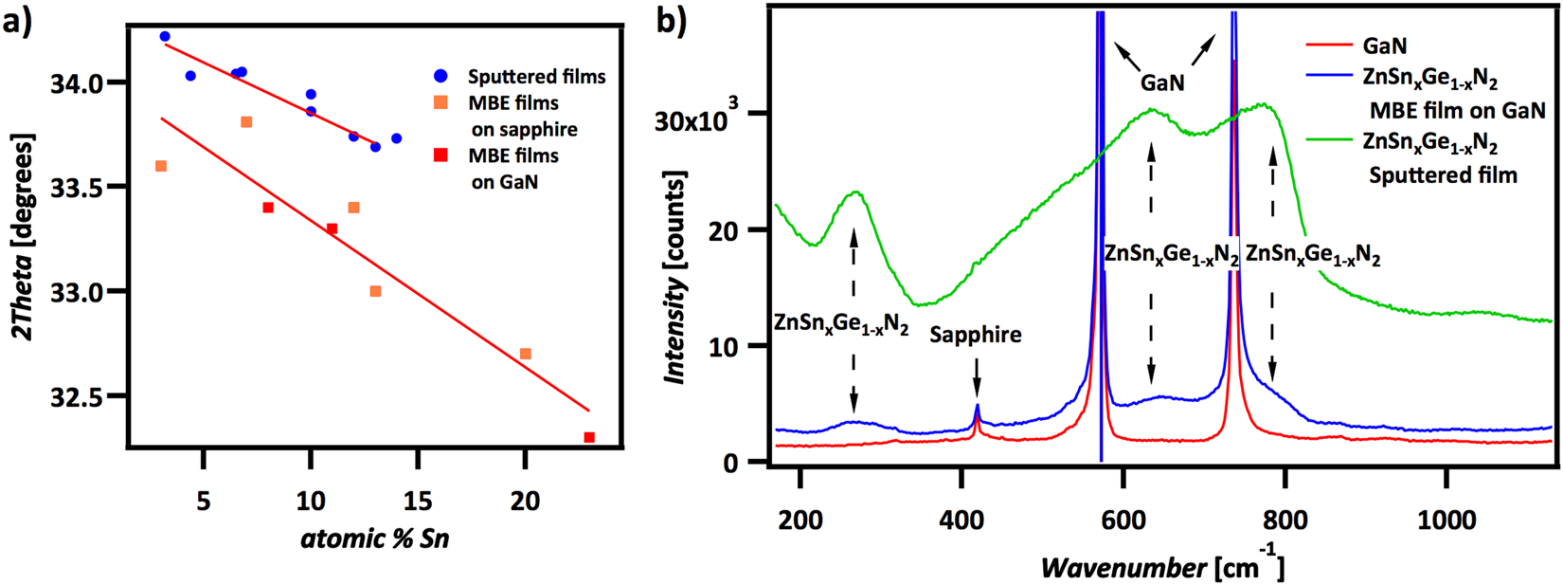
(**a**) Plot of ZnSn_x_Ge_1−x_N_2_ XRD peak positions vs tin concentration, for various stoichiomeries of ZnSn_x_Ge_1−x_N_2_ as indicated in the figure. Sputtered films^14^ exhibited a different shift compared to MBE films, potentially due to strain. (**b**) Raman spectra of a ZnSn_x_Ge_1−x_N_2_ MBE film compared to a sputtered film of similar stoichiometry (x ≈ 0.3). The broad peaks indicate lattice site disorder.

The strain present in films grown on substrates can cause deviations in lattice spacing, and consequently can produce deviations in XRD peak positions. Figure 3a compares the (002) peak positions for MBE films with the peak positions for sputtered films^7^. A wider spread in peak position was observed for MBE films relative to the spread for sputtered films, but the peak positions of the MBE-grown films still showed a linear trend with composition, in accordance with Vegard’s Law^15^. The difference in peak positions for MBE films and sputtered films at the various ZnSn_x_Ge_1−x_N_2_ stoichiometries can be attributed to differences in strain and epitaxy.

Although the positions of the (002) XRD peaks differed between MBE-grown and sputtered films, Raman spectroscopy revealed similar bonding within MBE films and sputtered films, respectively (Fig. 3b). The Raman spectra showed broad peaks at 270^−1^, 650 cm^−1^, and 760 cm^−1^ for both sputtered and MBE-grown ZnSn_x_Ge_1−x_N_2_ films, reflecting the material congruence. Raman spectroscopy probes bond vibrations, with sharp peaks expected for defined, periodic bond vibrations, unlike amorphous solids that instead exhibit broad features. The broad ZnSn_x_Ge_1−x_N_2_ peaks indicate that the bond vibrations probed are not well ordered, representing non-periodicity in the lattice^16^ that may result from random cation positioning^17–19^. Studies of plasma-assisted vapor-liquid-solid growth of ZnSnN_2_ at 485 °C resulted in similarly broad Raman peaks, with the broad peaks attributed to imperfect lattice ordering^16^ such as cation anti-site defects^18–20^.

Lattice-site disorder, point anti-site defects, and oxygen or other impurities may generate unwanted dopants or trap states not found in ideal computational predictions^17,20–22^. Additionally, surface states, strain, and grain boundaries, especially of 3D films grown on sapphire, influence electronic properties by bending bands, breaking band degeneracies, and inducing mobility changes^22,23^.

**Figure 4:**
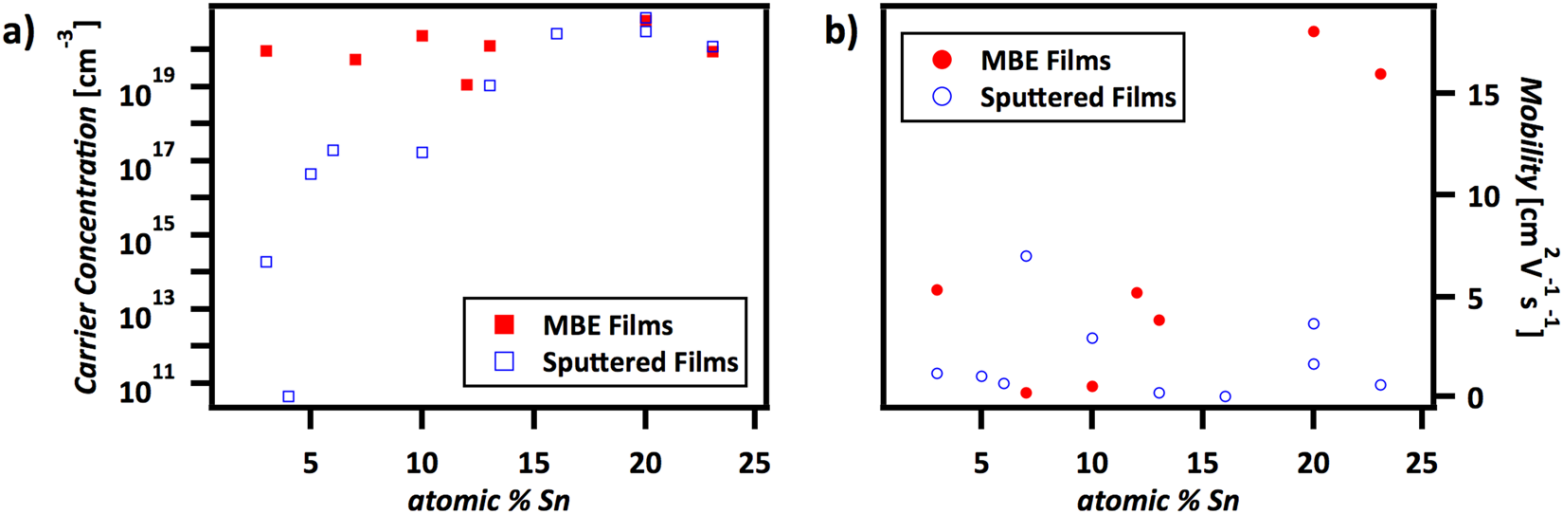
MBE ZnSn_x_Ge_1−x_N_2_ films on sapphire compared to sputtered films on sapphire for (**a**) carrier concentrations and (**b**) electronic mobilities. The stoichiometries for sputtered films were measured by EDS^14^, and the stoichiometries for MBE films were measured by XPS. Sputtered films were ~1 μm thick whereas MBE films were ~30 nm thick.

These imperfections may contribute to the difference in electronic properties between the nanocrystalline sputtered films and heteroepitaxial MBE films. Figure 4 shows the carrier concentrations and mobilities of the ZnSn_x_Ge_1−x_N_2_ MBE films grown on sapphire compared to the properties of the sputtered alloys. Sputtered alloys display an average mobility of ~2 cm^2^ V^−1^ s^−1^ (Fig. 4b). Furthermore, their carrier concentrations decrease from degenerate ZnSnN_2_ at 25% atomic Sn to non-degenerate low-tin ZnSn_x_Ge_1−x_N_2_. In contrast, epitaxial MBE films grown on sapphire showed degenerate carrier concentrations throughout the various compositions. Although high-tin-concentration MBE ZnSn_x_Ge_1−x_N_2_ and ZnSnN_2_ grown on sapphire exhibited a mobility of ~18 cm^2^ V^−1^ s^−1^, other compositions of MBE ZnSn_x_Ge_1−x_N_2_ samples grown on sapphire still had mobilities of <10 cm^2^ V^−1^ s^−1^.

Heteroepitaxial MBE films grown on GaN were probed for electronic mobility, but contributions ascribable to the GaN substrate did not allow measurement of the film properties. The GaN templates were >1 μm thick, whereas ZnSn_x_Ge_1−x_N_2_ alloy films did not exceed 50 nm in thickness. Slow growth rate limited the ability to obtain sufficiently thick ZnSn_x_Ge_1−x_N_2_ films to probe the bulk electronic properties of the 2D epitaxial films on GaN.

The epitaxial strain or surface contribution of the <50 nm thick MBE films on sapphire may be the cause of the ZnSn_x_Ge_1−x_N_2_ degeneracy and mobility change. The ZnSn_x_Ge_1−x_N_2_ carrier concentrations should decrease with increasing Ge content, in accord with the trend observed in the measured bulk carrier concentrations of sputtered films (~1 μm thick). The resulting degeneracy of the MBE-grown ZnSn_x_Ge_1−x_N_2_ thin films, for which the limited MBE film thickness enables surface effects to dominate measurements relative to bulk properties, potentially arises from excess mobile carriers from surface states or surface oxides. High carrier populations reduce the mobility, but the increased mobility of MBE films with increasing tin content (toward ZnSnN_2_) may be attributed to the epitaxial strain, as strain can influence the carrier mobility and the observed ZnSn_x_Ge_1−x_N_2_ strain increases with increasing tin content^23,24^. Thus, strain and surface- state effects likely dominate the electronic results.

Even at the current growth temperature settings of 250 °C, the high mobilities of high-tin-concentration MBE ZnSn_x_Ge_1−x_N_2_ on sapphire may have resulted from improved crystallinity or strain effects. However, the relatively low temperature of 250 °C can limit the potential to obtain a perfectly ordered crystal. Sharp, well-defined Raman peaks have been observed from crystalline ZnGeN_2_ needles and platelets grown at 750 °C and higher temperatures^18,19,25,26^, suggesting that higher cation ordering might be achieved through the MBE growth of ZnSn_x_Ge_1−x_N_2_ samples at higher temperatures than used herein, if thermal expansion coefficients allow for such on suitable substrates. Investigations utilizing higher temperature growth may reduce point defects and strain, with enhancement of optoelectronic properties, and enable the development of material suitable for use in high-performance devices.

In summary, heteroepitaxial MBE growth of ZnSn_x_Ge_1−x_N_2_ films on c-plane sapphire and hexagonal GaN templates has yielded improved crystallinity and electronic mobilities of ZnSn_x_Ge_1−x_N_2_ relative to sputtered films. The MBE films, while maintaining tunability and phase stability throughout the alloy series (0 ≤ x < 1), form cohesive, 2D films on GaN. TEM showed that low-substrate-temperature (250 °C) growth on GaN templates produced smooth, epitaxial, single-crystalline-quality films. Thicker films on GaN may allow electronic measurements of these improved 2D films. Growth at higher temperatures may further reduce point defects and enhance the optoelectronic properties, assisting the development of material for applications in high-performance semiconductor devices.

### Data Availability

The datasets generated during and/or analyzed during the current study are available from the corresponding author on reasonable request.

## Electronic supplementary material

Supplementary Info
