# Supplementary material for: Growth of Epitaxial ZnSnxGe1−xN2 Alloys by MBE
Source: Sci Rep. 2017 Sep 20;7:11990. doi: 10.1038/s41598-017-12357-9 (PMC5607306; doi:10.1038/s41598-017-12357-9)
Supplement: Supplementary file 1 — Supplementary Info [file 41598_2017_12357_MOESM1_ESM.pdf]

Supplemental Information

Growth of Epitaxial $ZnSn_xGe_{1-x}N_2$ Alloys by MBE

Amanda M. Shing, Yulia Tolstova, Prof. Nathan S. Lewis*, Prof. Harry A. Atwater**

Debye-Scherrer Approximation for Grain Size of Sputtered Films

The Debye-Scherrer approximation for grain size is given by the general equation listed below, where 0.9 is the shape factor, λ is the X-ray wavelength, β is full width at half-maximum of the XRD peak, and θ is the Bragg angle in radians. Cu Kα radiation (λ=1.54056 Å) was used. No strain broadening was accounted for.

$t = 0.9\lambda/\beta\cos\theta$ (Equation 1)

Equation 1 was used to perform grain size estimates for sputtered thin films on substrates. The equation assumes a powder sample, whereas we used sputtered films that were adhered to substrates. Therefore, the size estimate may not be accurate. However, cross-sectional SEM and TEM images of sputtered samples on substrates do not show detectable grains on the order of 200 nm or larger, implying that grain sizes are smaller than 200 nm.

Fabrication Details for $ZnSn_xGe_{1-x}N_2$ MBE Films

C-plane sapphire or GaN-template-on-sapphire substrates were loaded without pre-treatment into an SVT Associates Nitride MBE chamber. Chamber base pressures were $< 2\times10^{-7}$ torr. Substrates were heated to 250 °C for 2 hrs. before a nitrogen-plasma cleaning. The nitrogen plasma was delivered to each substrate by an HD-50 Oxford Instruments nitrogen-atom source at approximate chamber pressures of 3 $\times10^{-5}$ Torr to 5×10^{-5} Torr and 300W power. While rotating, each substrate was cleaned for ~10 min before beams from separate elemental Zn, Sn, and Ge Knudsen cells, employed at discrete temperatures, were introduced. Table S1 lists the parameters for the MBE growths, including substrate temperatures, Knudsen cell temperatures, and nitrogen plasma parameters.

Table S1. Parameters for MBE growths and stoichiometries measured by XPS. Rows noted with asterisks were grown on GaN substrates, whereas all others were grown on sapphire substrates.

$T_{substrate}$ [C]	Chamber Pressure N_2 [Torr]	RF Power [W]	T_{Zn} [C]	T_{Sn} [C]	T_{Ge} [C]	%N	%Zn	%Sn	%Ge	
250	3-5x10^{-5}	300	275	900	-	52	28	20	-	
250	3-5x10^{-5}	300	275	900	-	56	21	23	-	*
250	3-5x10^{-5}	300	275	900	-	59	14	28	-	a)
250	3-5x10^{-5}	300	275	900	775	52	26	20	2	
250	3-5x10^{-5}	300	275	860	1175	49	30	13	8	
250	3-5x10^{-5}	300	275	860	1200	52	26	12	9	
250	3-5x10^{-5}	300	275	830	1225	51	25	7	18	

250	3-5x10^{-5}	300	275	810	1250	50	22	3	25	
250	3-5x10^{-5}	300	275	830	1225	50	22	8	20	*
250	3-5x10^{-5}	300	275	845	1215	51	18	10	21	
250	3-5x10^{-5}	300	275	845	1215	53	22	11	14	*
250	3-5x10^{-5}	300	275	845	1215	52	17	12	19	

[a] After growth, the Knudsen cell shutter did not close within 30 seconds of the other sources.

After growth, in-situ RHEED patterns were obtained from the film surfaces. The electron beam was held at 9 kV, 2.5 A filament current, 0.5 A emission current, and ~ 6.3 kV objective voltage. Samples were then transferred to atmosphere and sections were cut off to perform X-ray photoelectron spectroscopy (XPS). Table 1 displays the resulting XPS stoichiometries. The error in XPS stoichiometry determination is generally ± atomic 10%, and the technique only probes the first ~10nm of the film. Adventitious carbon and oxygen signals were not included in the reported results. Although some surface oxidation may be present, the precise amount could not be distinguished from adventitious material. Argon-ion sputter cleaning of samples preferentially sputtered nitrogen from the compound semiconductor, leaving it with more metallic character. Other non-destructive characterization methods, such as Energy Dispersive Spectroscopy (EDS) or X-Ray Fluorescence (XRF), on the <50 nm thin films were inconclusive, as they incorporated oxygen or nitrogen originating from the substrates.

Thru-film oxidation was not assumed because studies of $ZnSn_xGe_{1-x}N_2$ films that were made by reactive radio-frequency sputtering and probed by energy dispersive spectroscopy (EDS) at 10kV (~200nm penetration depth [b]) displayed compositional stability and consistent electrical mobility measurements after a year. Only thicker MBE films would facilitate the certainty in the extent of oxidation.

Sample cross-sections analyzed by transmission electron spectroscopy (TEM) used an electron-beam energy of 300 keV and a camera length of 250 mm. A polycrystalline gold standard at the same energy and camera length were used for calibrating the distances and calculating the $ZnSn_xGe_{1-x}N_2$ lattice parameters. Lattice parameters were calculated from the TEM selected area diffraction. The Zn-IV-Nitride lattices are orthorhombic, thus geometric calculations were performed to obtain the orthorhombic *a* and *c*. The *b* lattice parameters could not be obtained because the films were oriented.

Hall measurements of $ZnSn_xGe_{1-x}N_2$ films on sapphire and GaN were performed using ohmic In contacts.

[b] http://www.globalsino.com/EM/page4795.html

RHEED of $ZnSn_xGe_{1-x}N_2$ 3D Growth on Sapphire

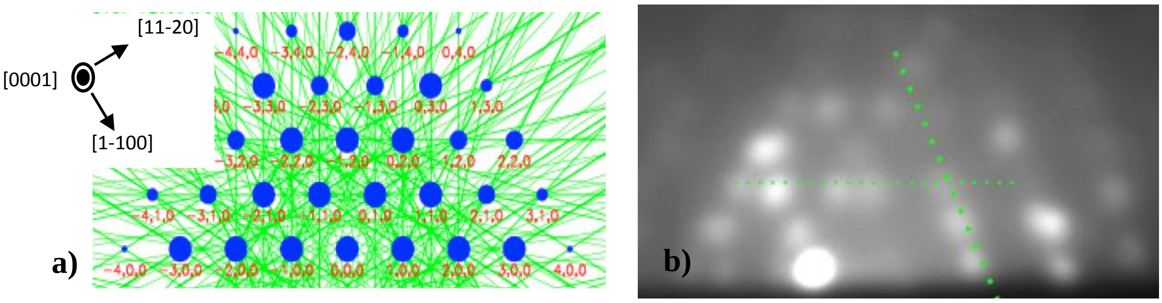

Figure S1 a) Simulated EMAPS (Reference: Zuo and Mabon, Micosc. Microanal. 2004) of selected area transmission diffraction pattern for a wurtzite GaN lattice compared to b) $ZnSn_xGe_{1-x}N_2$ RHEED of 3D island growth, represented by the spotted pattern, on sapphire. [Reference 11]

Epitaxial 3D island growth of $ZnSn_xGe_{1-x}N_2$ on sapphire was observed. [Reference 11] The epitaxial nature was seen from the Pendellosung oscillations of the (002) $ZnSn_xGe_{1-x}N_2$ XRD peak. SI Figure 1b shows the $ZnSn_xGe_{1-x}N_2$ RHEED image as spots with similar symmetry as the simulated hexagonal GaN pattern for transmission diffraction. Matching symmetry of diffraction patterns to hexagonal GaN is expected for wurtzite $ZnSn_xGe_{1-x}N_2$ 3D islands because the electron beam transmits through the peak of the crystal island to create the diffraction pattern. [Reference 11]

Table S2. Measured interfacial lattice parameters calculated from SAED of MBE films on GaN substrates. MBE $ZnSnN_2$ films are strained on GaN and average lattice parameters are given. Films are oriented on the substrate, so only *a* and *c* parameters can be calculated.

	a [nm]	b [nm]	c [nm]
$ZnSnN_2$ on GaN	1.122	-	1.039
$ZnSn_xGe_{1-x}N_2$ on GaN	0.6592	-	0.5291

Band Gap Tunability

Ellipsometry was performed with a JA Woollam Alpha-SE ellipsometer and the data were modeled with Complete-Ease Software using a wavelength-by-wavelength layer on top of an alumina substrate layer. Using the extinction coefficients, absorption coefficients were calculated to be on the order of 10^5 cm^{-1}, highlighting good absorption properties, similar to known thin-film absorbers like CdTe. Extinction coefficients were transformed to absorption coefficients with Equation 2, where α is the absorption coefficient, *k* is the extinction coefficient, and λ is the probing wavelength. The data were then fitted using the Tauc method for direct band gaps. The x-intercepts for squared absorption coefficients were collected and plotted by composition.

$\alpha=4\pi k/\lambda$ (Equation 2)

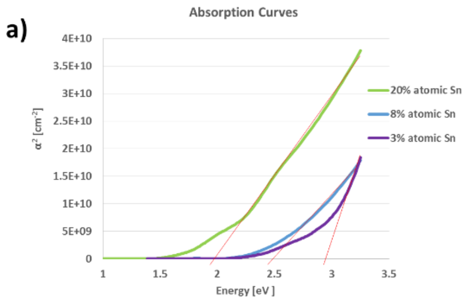

b)

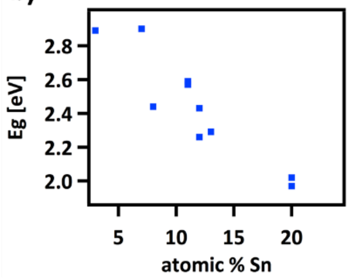

Figure S2 a) Direct band gap Tauc plot for $ZnSn_xGe_{1-x}N_2$ samples of different compositions with linear fits. **b)** Compiled Tauc-derived band gaps over various stoichiometries for the MBE $ZnSn_xGe_{1-x}N_2$ thin films show that the band gap increases with increasing Ge content and decreasing Sn content, displaying tunability.
